# First Detection of Antibodies Specific to Crimean-Congo Hemorrhagic Fever Virus in Rural Populations of Gabon

**DOI:** 10.4269/ajtmh.24-0054

**Published:** 2024-07-23

**Authors:** Linda Bohou Kombila, Solène Lerolle, Illich Manfred Mombo, Neil-Michel Longo-Pendy, Danielle Koumba Mavoungou, Gaël Darren Maganga, Francois-Loïc Cosset, Jessica Vanhomwegen, Christina Deschermeier, Eric Maurice Leroy, Vincent Legros, Nadine N’dilimabaka, Pierre Becquart

**Affiliations:** ^1^ Département de Virologie, Unité Emergence des Maladies Virales, Centre Interdisciplinaire de Recherches Médicales de Franceville, Franceville, Gabon;; ^2^ Ecole Doctorale Régionale d’Afrique Centrale en Infectiologie Tropicale, Université des Sciences et Techniques de Masuku, Franceville, Gabon;; ^3^ Centre International de Recherche en Infectiologie, Université de Lyon, Université Claude Bernard Lyon 1, l’Institut National de la Santé et de la Recherche Médicale, U1111, Centre National de la Recherche Scientifique, UMR5308 École Normale Supérieure de Lyon, Lyon, France;; ^4^ Unité de Recherche en Ecologie de la Santé, Centre Interdisciplinaire de Recherches Médicales de Franceville, Franceville, Gabon;; ^5^ Institut National Supérieur d’Agronomie et de Biotechnologies, Université des Sciences et Techniques de Masuku, Franceville, Gabon;; ^6^ Cellule d’Intervention Biologique d’Urgence, Institut Pasteur de Paris, Paris, France;; ^7^ Panadea Diagnostics GmbH, Hamburg, Germany;; ^8^ Maladies Infectieuses et Vecteurs, Ecologie, Génétique, Evolution et Contrôle, Université Montpellier, Institut de Recherche pour la Développement, Centre National de la Recherche Scientifique, Montpellier, France;; ^9^ Campus Vétérinaire de Lyon, VetAgro Sup, Université de Lyon, Marcy-l’Etoile, France;; ^10^ Département de Biologie, Faculté des Sciences, Université des Sciences et Techniques de Masuku, Franceville, Gabon

## Abstract

Crimean-Congo hemorrhagic fever (CCHF) is a tick-borne viral disease with a mortality rate reaching up to 40% in humans. Currently, CCHF affects three continents: Asia, Europe, and Africa. An increase in confirmed cases in Africa has been observed since 2000. In Central Africa, several countries have reported the circulation of CCHV virus (CCHFV). However, in Gabon, there is a lack of recent data on the circulation of the virus in the Gabonese population. To provide an overview of the epidemiological situation in Gabon, we tested 3,081 human serum samples collected between 2005 and 2008 in villages throughout the country for anti-CCHFV antibodies. Using a double-antigen ELISA kit, our study found 15/3,081 samples positive for CCHFV. These positive samples were also tested using the Blackbox CCHFV IgG kit and the Luminex technique. These analyses confirmed seven and four positives for the Blackbox CCHFV IgG kit and the Luminex technique, respectively. This study suggests low circulation of CCHFV in the rural human population of Gabon. Competent authorities must survey CCHFV to identify and prevent clinical cases in the human population.

## INTRODUCTION

Crimean-Congo hemorrhagic fever (CCHF) is a severe tick-borne zoonosis caused by CCHF virus (CCHFV; genus *Orthonairovirus*, family *Nairoviridae*).^1^ Specific antibodies against CCHFV have been found in both domestic and wild vertebrates. Although different animal species can be infected with CCHFV, they do not develop any clinical illness.^2^ Humans appear to be the only host susceptible to severe clinical disease. Subclinical infection is frequently observed in human cases after exposure to the virus. However, vascular leakage, multiorgan failure, shock, and severe bleeds leading to coma or death have been reported to occur in 10–40% of patients.^3^^–^^5^

Infection in humans typically occurs via the bite of an infected tick but can also result from direct or indirect contact with contaminated biological fluids, organs, and tissues of viremic animals or people.^6^ Crimean-Congo hemorrhagic fever is considered a major threat to human health and an important, yet neglected disease with severe public health and socioeconomic consequences. The World Organization for Animal Health and the WHO prioritized CCHFV as one of the leading emerging and reemerging pathogens of interest.

The global distribution of CCHF corresponds closely to that of its main arthropod vector, the ixodid ticks belonging to the genus *Hyalomma*. The first identified cases occurred at the end of World War II in Crimea. Today, the virus is present in the Middle East, eastern and southern Europe, western Asia, and Africa, and a total of 19 countries have reported CCHF infections.^7^ The first documented cases of infection in Africa date back to 1956 in the Democratic Republic of Congo (DRC).^7^ Since then, other countries in Central Africa have reported human cases of CCHF: the Central African Republic, Cameroon, Equatorial Guinea, and Republic of Congo. The recent human case described in central Africa dates from 2008 in the DRC.^8^ In Gabon, despite the lack of recent studies on the circulation of CCHFV, indirect evidence has demonstrated circulation of the virus in the country. Neutralizing antibodies against the viral glycoprotein of CCHFV were detected in bats (order: Chiroptera) collected from 2005 to 2009 in the northeast of Gabon.^7^^,^^9^ In addition, antibodies against CCHFV were detected in 2018 in samples from pygmies in Cameroon, a country bordering Gabon in the north.^10^ To obtain information on the circulation of the virus in the country, we analyzed the biobank of serum samples provided by the studies of Becquart and collaborators who carried out large serosurveys in rural areas of Gabon.^11^ The aim of our study was to assess the circulation of CCHFV in the Gabonese rural population.

## MATERIALS AND METHODS

### Study population and blood sample collection.

For this study, we used serum samples from a previous serosurvey conducted by Becquart et al.^11^ in Gabon from June 2005 to September 2008. The samples were collected from 3,081 individuals in 199 randomly selected villages for serological studies of hemorrhagic fever diseases like Ebola and Marburg. This survey focused on rural villages with fewer than 300 inhabitants located in the nine administrative regions of Gabon: Estuaire, Haut-Ogooué, Moyen-Ogooué, Ngounié, Nyanga, Ogooué-Ivindo, Ogooué-Lolo, Ogooué-Maritime, and Woleu-Ntem. All permanent residents aged more than 15 years were invited to participate and provided blood samples.

### Cell culture.

Huh7.5 hepatocarcinoma cells stably expressing firefly luciferase (fLuc) were grown in Dulbecco’s modified Eagle’s medium (Gibco, Grand Island, NY) supplemented with 10% fetal calf serum, 100 U/mL of penicillin, and 100 µg/mL streptomycin (Gibco). All cells were grown in a 37°C and 5% CO_2_ incubator. The cells were regularly tested for mycoplasma contamination.

#### Antibodies.

Mouse monoclonal antibodies targeting CCHFV strain Ibar10200 anti-PreGc (clone 30F7) was obtained from the Joel M. Dalrymple-Clarence J. Peters USAMRIID Antibody Collection through BEI Resources, National Institute of Allergy and Infectious Diseases, NIH. Anti-vesicular stomatitis virus protein G (anti-VSV-G) 41A.1 was described previously.^12^ A mouse IgG isotype (catalog no. 31903) was obtained from Invitrogen (Carlsbad, CA).

### Plasmids and constructs.

The constructs encoding wild-type CCHFV strain IbAr10200 V5-tagged L polymerase (pCAGGS-V5-L), CCHFV nucleoprotein (pCAGGS-NP), CCHFV-specific nanoluciferase (nLuc)-expressing minigenome (pSMART-LCK_T7-vL-nLuc), CCHFV glycoprotein precursor (pCAGGS-GPC), and T7 RNA polymerase (pCAGGS-T7) were previously described.^13^^,^^14^ psPAX2 and phCMV (expression vector)-VSV-G encoding the G glycoprotein of VSV are kind gifts from Didier Trono and Jane Burns, respectively. pHR′-CMV-nLuc-WPRE (woodchuck post-transcriptional regulatory element) was previously described.^15^

### Detection of CCHFV antibodies.

The detection of antibodies against CCHFV was performed by ELISA using the kit ID Screen^®^ CCHF double antigen for multispecies (Innovative Diagnostics, Grabels, France), according to the manufacturer’s instructions. This kit was developed for the detection of anti-CCHFV nucleoprotein antibodies in cattle, goats, sheep, or other susceptible species, including humans. It has a specificity and sensitivity of 100% and 98.9%, respectively.^16^ It has been used recently in several studies for antibody testing in human populations.^17^^–^^20^

Samples positive by ELISA were tested with the Blackbox CCHFV IgG ELISA kit (EVAg, Hamburg, Germany) targeting the CCHFV nucleoprotein according to the manufacturer’s instructions. This commercial kit demonstrated 100% specificity and sensitivity in a test of human samples.^21^

In addition, samples that tested positive by ELISA were screened by a microsphere-based multiplex immunoassay (MMIA; Luminex) for IgG antibodies specific to CCHFV nucleoprotein or glycoprotein. A sample is considered seropositive if the measured median fluorescent intensity value is above the cutoff for at least one of the target proteins. The cutoff was calculated from the following formula: cutoff = mean of seronegative values + 3SD.

### Seroneutralization assays using the CCHFV tc-VLP assay.

We used a reverse genetics approach to produce nLuc-expressing CCHFV virus-like particles, which are transcription- and entry-competent virus-like particles (tc-VLPs). Briefly, a total of 1.2 × 10^6^ Huh7.5 cells were seeded in a 10-cm dish and transfected with 1.2 µg of pCAGGS-NP, 3 µg of pCAGGS-GPC, 3.6 µg of pCAGGS-V5-L, 3 µg of pSMART-LCK_T7-vL-nLuc, and 3 µg of pCAGGS-T7, using GeneJammer transfection reagent (Agilent). At 6 hours posttransfection, the medium was changed, and tc-VLP-containing supernatants were harvested 72 hours later, cleared by centrifugation for 5 minutes at 750 × *g*, filtered through 0.45-µm-pore-size filters, and stored aliquoted at −80°C. Moreover, we produced nLuc-expressing VSV-G-pseudotyped lentiviruses. A total of 2.4 × 10^6^ HEK293T cells were seeded in a 10-cm dish and transfected with 2 µg of phCMV-VSV-G, 8 µg of psPAX2, and 8 µg of pHR′-CMV-nLuc-WPRE plasmids using calcium phosphate precipitation. At 16 hours posttransfection, the medium was changed, and supernatants were harvested 24 hours later, filtered through 0.45-µm-pore-size filters, and stored aliquoted at −80°C.

Neutralization assays were performed in a blind manner. For neutralization assays, nLuc tc-VLPs or nLuc VSV-G-pseudotyped lentiviruses were incubated with a 50-fold dilution of serum for 1 hour at 37°C. For the C39 serum, 2-fold serial dilutions from 1/200 to 1/25 were also tested. Then, the viruses were used to infect Huh7.5 cells stably expressing firefly luciferase (fLuc) seeded in 96 well-plates (2 × 10^4^ cells). Three hours postinfection, the inoculum was removed, the cells were washed with phosphate-buffered saline, and the medium was replaced with fresh medium. The cells were incubated for 24 hours at 37°C before lysis with 1× passive lysis buffer (Promega), and reporter activities were measured with a Dual-Glo luciferase assay system (Promega), in accordance with the manufacturer’s instructions, and a Mithras LB 940 system. Serum samples from CCHFV-unexposed humans from France (from healthy blood donors and from patients diagnosed with severe acute respiratory syndrome coronavirus 2 or hepatitis C virus infection) and mouse IgG (10 µg/mL) were used as negative controls. An anti-Gc neutralizing antibody (clone 30F7, 10 µg/mL) or anti-VSV-G neutralizing antibody were used as positive controls. The relative luminescence units (RLU) signal of nLuc was normalized to the RLU signal of fLuc. Then, results were expressed as the percentage of infection efficiency relative to that of the IgG control set at 100%. The experiment was performed twice in technical triplicate, except for the serial dilution of the C39 sample, which was performed once in technical triplicate.

## STATISTICAL ANALYSES

Associations between the detection of antibodies (positive sample highlighted from the kit ID Screen CCHF) and age, sex, environment, and the practice of hunting were assessed through binomial generalized linear models. The environments of the villages were coded in three classes, savannah, lagoon, and forest, with savannah having been chosen as the base category.

## RESULTS

### Description of the population.

**Figure 1.**
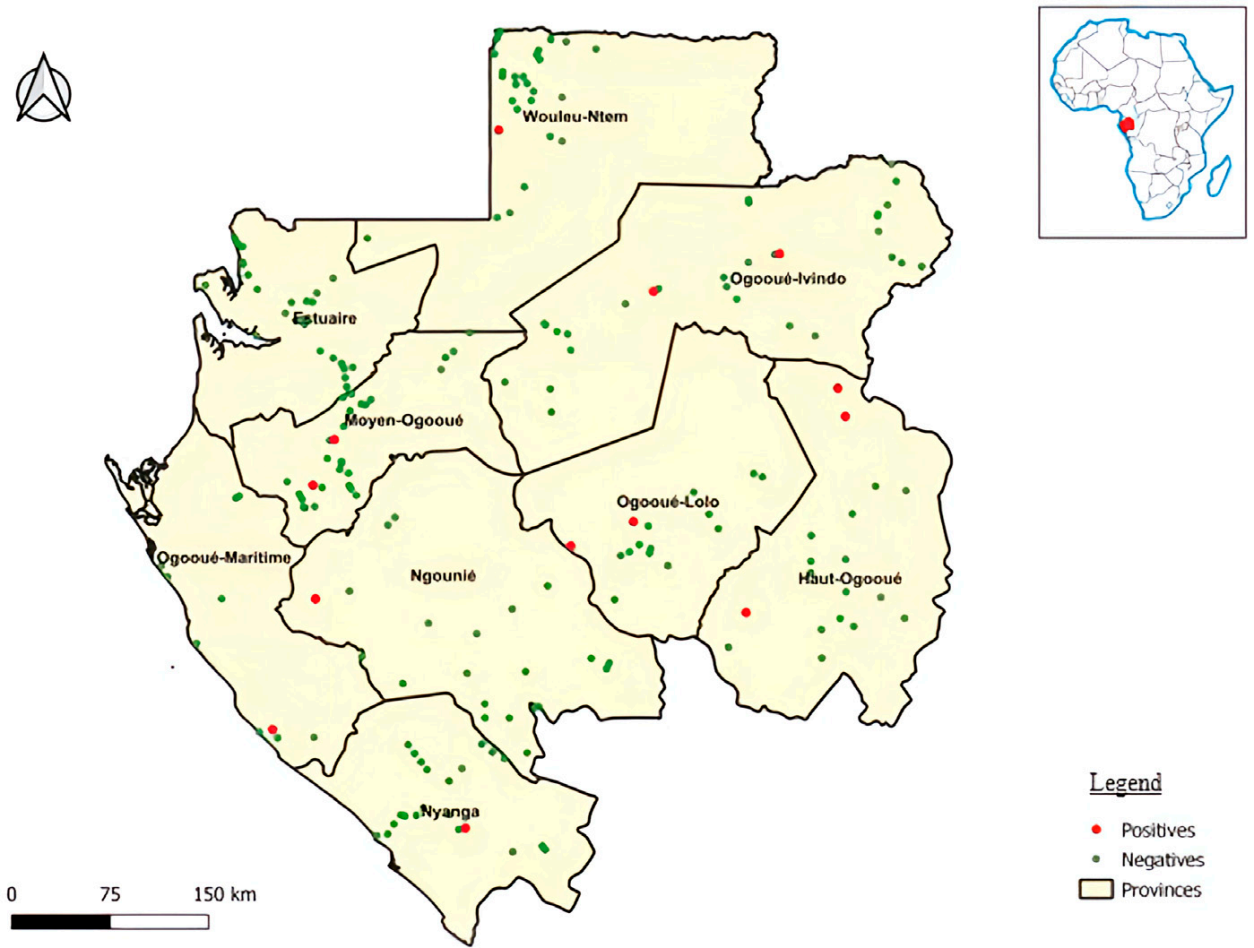
Distribution of sampling collecting sites. Villages with positive and negative samples are indicated by red and green dots, respectively.

A total of 3,081 serum samples from individuals in 199 villages throughout Gabon were analyzed (Figure 1; Supplemental Table 1). The study population according to sex included 1,461 men and 1,620 women. The ages ranged from 16 to 90 years old (mean age, 47.3 ± 14.2 years). The most represented type of environment was forest, with 2,323 people living in this ecosystem. Agriculture was the most common activity carried out by the people, with 2,184 individuals (Supplemental Table 1).

### CCHFV-specific antibody seroprevalence.

**Figure 2.**
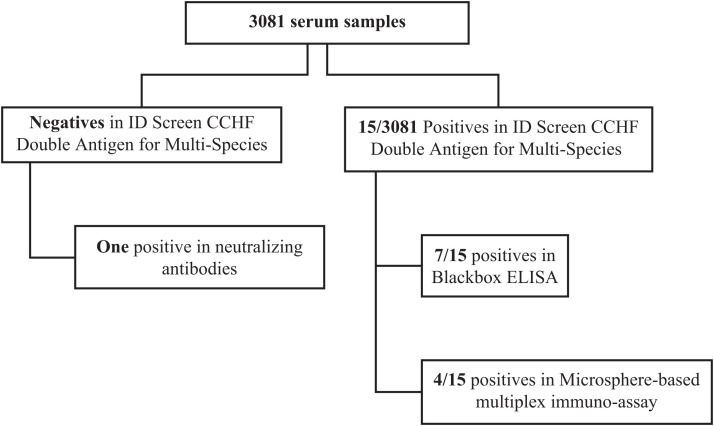
Tests and results of the study.

Anti-CCHFV antibodies were detected with the kit ID Screen CCHF double antigen for multispecies in 15 individuals (seven men and eight women) scattered throughout the country, with no apparent link to each other, with an overall seroprevalence rate of 0.5% (Figure 1). The Blackbox CCHFV IgG ELISA confirmed 7 of 15 double-antigen ELISA positives. Analysis with the MMIA technique confirmed 4 of 15 double-antigen ELISA positives. Two were positive for nucleoprotein and glycoprotein. One sample was positive for the three tests. The results are summarized in Figure 2.

The results of the statistical analysis, based on the positive samples highlighted from the kit ID Screen CCHF, showed no significant risk factors (Supplemental Table 2).

### Detection of CCHFV-neutralizing antibodies.

**Figure 3.**
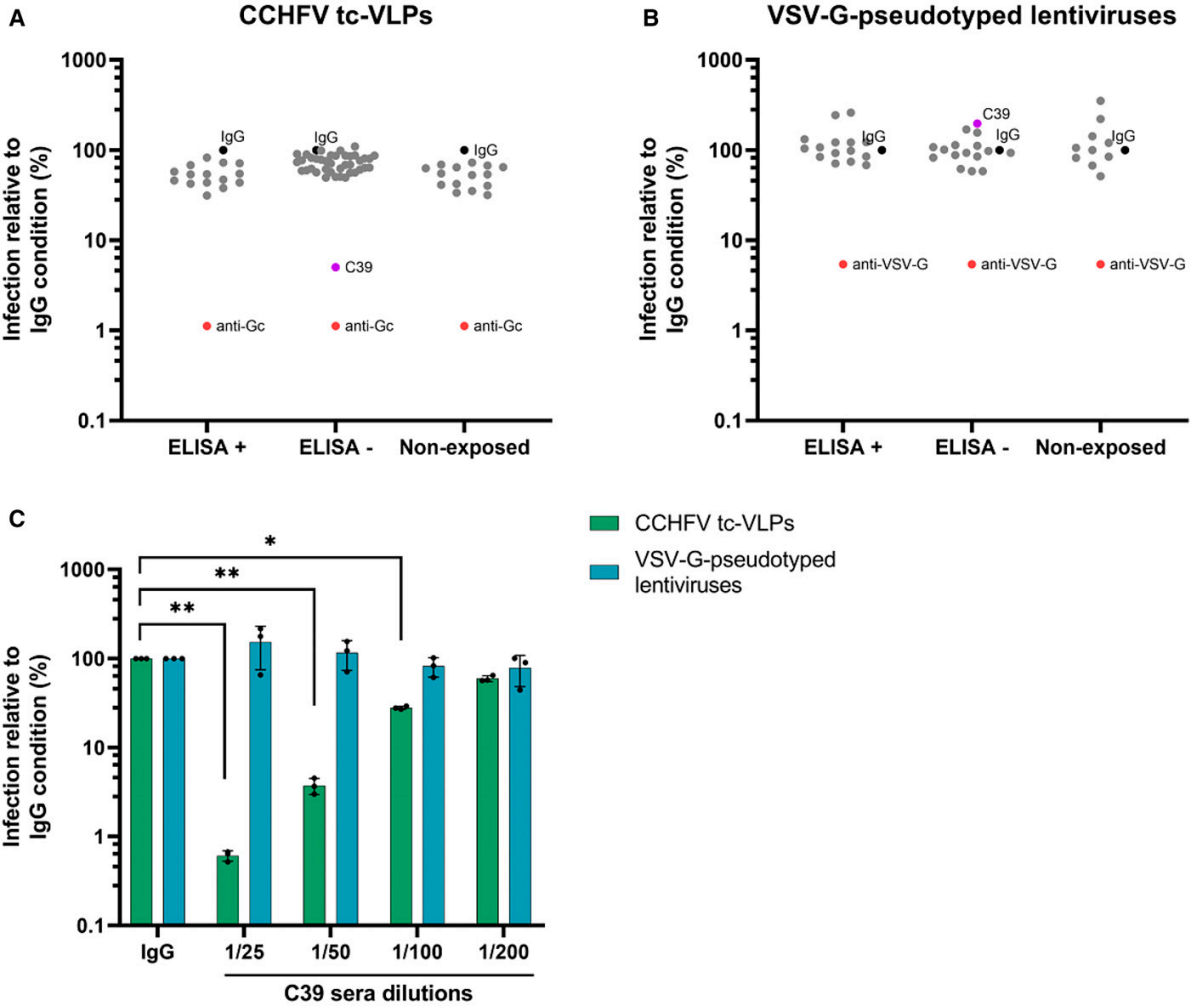
Seroneutralization assays using the Crimean-Congo hemorrhagic fever virus (CCHFV) transcription- and entry-competent virus-like particle (tc-VLP) system or vesicular stomatitis virus G protein (VSV-G)-pseudotyped lentiviruses on selected ELISA samples. (**A**) ELISA-positive and -negative serum samples or nonexposed human control sera diluted 1/50 were incubated with CCHFV tc-VLPs expressing nanoluciferase prior to infection of Huh7.5 cells stably expressing firefly luciferase. Luminescence was read 24 hours postinfection. Results were normalized to the IgG control (black dot), and an anti-Gc neutralizing antibody (red dot) was used as a positive control. Data represent the mean of two independent experiments performed in technical triplicate. (**B**) The same procedure as that described for panel A was performed using a VSV-G-pseudotyped lentivirus-based neutralization assay. (**C**) The C39 serum sample was serially diluted (2-fold) from 1/200 to 1/25, and the same procedure as that described for panels A and B was performed. The mean and standard deviation of results of one experiment performed in technical triplicate are shown. Statistical significance was analyzed using a two-way analysis of variance with Dunnett’s multiple comparison test (*P <0.05; **P <0.01).

To identify CCHFV-neutralizing antibodies in patients and to confirm the results obtained with the ELISA tests, we performed seroneutralization assays using an in vitro assay for nLuc reporter gene-expressing CCHFV tc-VLPs.^22^ Fifty-five serum samples (from C1 to C55), of which 15 were ELISA-positive samples (C4, C8, C12, C14, C16, C21, C23, C31, C34, C35, C44, C47, C48, C52 and C54), were assessed for their neutralizing activity. Additionally, 16 samples from humans sampled in France (CCHFV nonexposed, negative controls) were included in the seroneutralization testing. Intriguingly, none of the ELISA-positive samples gave higher neutralization levels than the negative controls, in contrast to a monoclonal CCHFV-Gc-neutralizing antibody (Figure 3A). Strikingly, one ELISA-negative sample, namely C39, gave significantly higher neutralization than negative controls, in a dose-dependent manner (Figure 3A and C). To test the specificity of the neutralizing activity of the serum samples, we used a VSV-G-pseudotyped lentivirus-expressing nLuc reporter. None of the serum samples exhibited neutralizing activity, in contrast to a VSV-G-neutralizing monoclonal antibody, further confirming the specificity of the CCHFV tc-VLP assay (Figure 3B and C). Overall, we were not able to correlate the ELISA-positive samples using the seroneutralization assay. Moreover, the C39 ELISA-negative sample showed high seroneutralization levels, which could suggest that some CCHFV-positive individuals may not have been detected by ELISA.

## DISCUSSION

To our knowledge, this is the largest human serological survey of CCHFV conducted to date in Central Africa, and more particularly in Gabon, as it involved more than 3,000 samples from all Gabonese regions. The first study was carried out in 1987 on 213 inhabitants in a village in the Haut-Ogooué province in the southeast of the country.^23^ The second study was carried out in 1989 on 1,841 individuals from five cities in five different Gabonese provinces: Haut-Ogooué, Estuaire, Ogooué-Ivindo, Ngounié, and Ogooué-Maritime.^24^ We tested here 3,081 human serum samples collected between 2005 and 2008 in villages throughout the country for anti-CCHFV antibodies. Using a double-antigen ELISA kit, 15 samples were found positive for CCHFV (seroprevalence of 0.5%). These positive samples were then tested using the Blackbox CCHFV IgG kit and the Luminex technique. These analyses confirmed seven and four positives for the Blackbox CCHFV IgG kit and the Luminex technique, respectively. However, no positive samples could be confirmed by seroneutralization. Our results were consistent with studies conducted in five other Central African countries (Chad, Cameroon, Central African Republic, Republic of Congo, and Equatorial Guinea), which showed seroprevalence rates ranging from 0.0% to 0.7%.^18^^,^^24^ The results of seroneutralization raised intriguing discrepancies with ELISA assays. Indeed, we could surprisingly not confirm the positivity of any ELISA-positive sample by seroneutralization. This could be explained by the detection of false-positive samples by ELISA and/or a low specificity of this method, although the two ELISA kits used in this study yielded similar results, challenging this possibility. Of note, ELISAs and the seroneutralization assays detect different types of antibodies. Whereas the ELISAs used in this study detect CCHFV antibodies targeting CCHFV nucleoprotein, the seroneutralization assay detects CCHFV antibodies targeting CCHFV glycoproteins through their neutralizing epitopes. Additionally, previous studies showed that CCHFV-infected patients produce only low levels of neutralizing antibodies, and these levels strongly diminished 19 weeks postinfection.^25^ This could explain why a serum sample can score positive in a nucleoprotein-based ELISA but negative in a seroneutralization assay. Moreover, whereas the neutralization assay detects antibodies against CCHFV glycoproteins from the IbAr10200 strain, it is crucial to consider the prevalence of various CCHFV strains circulating in regions proximate to Gabon. Strains belonging to the African II and III genotypes circulate in Central Africa.^8^^,^^18^ IbAr10200 belongs to the Africa III genotype, but it cannot be excluded that another strain, which could escape the antibody-mediated neutralization of the IbAr10200-based seroneutralization assay, circulates in Gabon. Interestingly, the seroneutralization assay revealed a single ELISA-negative sample (C39), demonstrating dose-dependent neutralization of tc-VLPs. This sample neutralization activity was specific to CCHFV tc-VLPs and not to VSV-G-pseudotyped lentiviruses. This suggests that the ELISA tests might result in false-negative results due to low sensitivity or higher levels of anti-Gc antibodies than anti-nucleoprotein antibodies in this subject. Moreover, this strongly suggests that CCHFV might circulate in Gabon and that some patients developed neutralizing antibodies against this pathogen. However, it is important to note that although neutralizing assays ex vivo are widely used to assess the neutralization capacity of antibodies in sera, they may not entirely replicate the natural viral neutralization process occurring within the human body. This could elucidate instances where a sample registers positive in a neutralization test but fails to reflect genuine neutralization in vivo. Overall, these discrepancies underscore the critical importance of employing multiple testing methods and complementary assays to gain a comprehensive understanding of CCHFV circulation in Gabon and elsewhere.

There are three modes of transmission of CCHFV to humans: 1) by tick bite; 2) by contact with infected tissue or blood from recently slaughtered animals; and 3) by contact with the blood of a sick person during the viremic stage.^26^ There are no data in the literature on the circulation of CCHFV in ticks in Gabon, which could be responsible for the positive results observed in our study. Although ticks of the genus *Hyalomma,* known to be the principal vector and reservoir of CCHFV, have never been found to our knowledge in Gabon, studies have reported the presence of non-*Hyalomma* ixodid ticks described as a vector of CCHFV.^27^ A study conducted by Moubamba^28^ between 2004 and 2005 showed the presence of two species of ticks in dogs in Libreville (Gabon), including *Rhipicephalus sanguineus* and *Amblyomma variegatum*. Another survey carried out between 2009 and 2010 reported three species of ticks, *Amblyomma variegatum, Rhipicephalus decoloratus*, and *Rhipicephalus sanguineus,* collected from wild and domestic animals and from the environment, in several regions of Gabon.^29^ All these species could serve as a vector for the transmission of the virus to Gabonese populations. In addition, the presence of CCHFV-specific antibodies was found in bats collected between 2005 and 2008 in Gabon.^9^ Frugivorous bat species, such as *Rousettus aegyptiacus*, shelter in human dwellings and feed on fruits also consumed by humans.^30^ Moreover, in some Gabonese provinces, bats are also hunted and consumed by human populations.^31^ These factors could lead to several direct or indirect contacts between bats and human populations, enhancing the possibility of zoonotic transmission of CCHFV. Finally, our study reveals the presence of anti-CCHFV antibodies in people from the Ogooué-Ivindo province, where bats have been found to carry neutralizing antibodies to CCHFV.

### Study limitations.

Our study presents some limitations. Indeed, sample collection was done nearly 20 years ago. It is highly probable that the epidemiology of CCHFV has changed significantly since the sampling period. The very low seroprevalence observed does not necessarily reflect very low virus circulation in Gabon. Studies using recent samples are needed to obtain a more recent CCHF epidemiological situation in Gabon.

## CONCLUSION

This study would indicate a potential circulation of the virus in Gabon, which can be confirmed only by identifying a clinical case in humans or ticks infected with CCHFV.

This immune detection of the causal agent in humans should alert public authorities to the need to set up a surveillance system for this arbovirus, which poses health risks for the public. In addition, the search for vectors would make it possible to identify potential contributors to the spread of CCHFV in humans.

## Supplemental Materials

10.4269/ajtmh.24-0054 Supplemental Materials
