# Supplementary material for: First Detection of Antibodies Specific to Crimean-Congo Hemorrhagic Fever Virus in Rural Populations of Gabon
Source: Am J Trop Med Hyg. 2024 Jul 23;111(4):880–6. doi: 10.4269/ajtmh.24-0054 (PMC11448543; doi:10.4269/ajtmh.24-0054)
Supplement: Supplemental Materials [file tpmd240054.SD1.pdf]

Table S1. Prevalence rates by sex, age groups, provinces, activities and environments.

Categories	Positives (n/N)	Prevalence rates (%)
**Sex**		
Men	7/1461	0.48
Women	8/1620	0.49
**Age groups**		
16-25	0/293	0.00
26-34	2/381	0.52
35-43	2/514	0.39
44-52	2/575	0.35
53-61	4/837	0.48
≥62	5/481	1.04
**Provinces**		
Estuaire	0/266	0.00
Haut-Ogooué	3/358	0.84
Ogooué-Maritime	1/178	0.56
Ngounié	3/295	1.02
Nyanga	1/375	0.27
Ogooué-Ivindo	2/407	0.49
Ogooué-Lolo	2/396	0.51
Moyen-Ogooué	2/433	0.46
Woleu-Ntem	1/373	0.27
**Activities**		
Farmers	9/2184	0.41
Hunters	2/329	0.61
Civil servants	3/179	1.68
Shopkeepers	0/60	0.00
Students	0/28	0.00
Others	1/160	0.63
None	0/141	0.00
**Environment**		
Forest	12/2323	0.52
Savannah	1/374	0.27
Lagoon	2/384	0.52

Table S2. Statistical analysis

Categories	**odds-ratio (95% CI)**	**P-value**
Ecosystem - Savannah	base	
Ecosystem - Lagoon	1.953 (0.176 - 21.62)	0.59
Ecosystem - Forest	1.936 (0.251 - 14.93)	0.53
Age (10 years increment)	1.462 (0.975 - 2.193)	0.053
Sex (Male)	0.970 (0.351 - 2.682)	0.95
Hunter	1.675 (0.579 - 4.841)	0.35

Legend : odds ratios, 95% confidence intervals (CI) and P-values, computed from binomial regression

Age : odds-ratio for a 10 years increment.

Sex : odds-ratio for male versus female.
